# On the Action of 5-Amino-Salicylic Acid and Sulfapyridine on *M. avium* including Subspecies *paratuberculosis*

**DOI:** 10.1371/journal.pone.0000516

**Published:** 2007-06-13

**Authors:** Robert J. Greenstein, Liya Su, Azra Shahidi, Sheldon T. Brown

**Affiliations:** 1 Department of Surgery, VA Medical Center, Bronx, New York, United States of America; 2 Laboratory of Molecular Surgical Research, VA Medical Center, Bronx, New York, United States of America; 3 Microbiology, VA Medical Center, Bronx, New York, United States of America; 4 Infectious Diseases, VA Medical Center, Bronx, New York, United States of America; The Institute for Genomic Research, United States of America

## Abstract

**Background:**

Introduced in 1942, sulfasalazine (a conjugate of 5-aminosalicylic acid (5-ASA) and sulfapyridine) is the most prescribed medication used to treat “inflammatory” bowel disease (IBD.) Although controversial, there are increasingly compelling data that *Mycobacterium avium* subspecies *paratuberculosis* (MAP) may be an etiological agent in some or all of IBD. We have shown that two other agents used in the therapy of IBD (methotrexate and 6-MP) profoundly inhibit MAP growth. We concluded that their most plausible mechanism of action is as antiMAP antibiotics. We herein hypothesize that the mechanism of action of 5-ASA and/or sulfapyridine may also simply be to inhibit MAP growth.

**Methodology:**

The effect on MAP growth kinetics by sulfasalazine and its components were evaluated in bacterial culture of two strains each of MAP and *M. avium*, using a radiometric (^14^CO_2_ BACTEC®) detection system that quantifies mycobacterial growth as arbitrary “growth index units” (GI). Efficacy data are presented as “percent decrease in cumulative GI” (%−ΔcGI).

**Principal Findings:**

There are disparate responses to 5-ASA and sulfapyridine in the two subspecies. Against MAP, 5-ASA is inhibitory in a dose-dependent manner (MAP ATCC 19698 46%−ΔcGI at 64 µg/ml), whereas sulfapyridine has virtually no effect. In contrast, against *M. avium* ATCC 25291, 5-ASA has no effect, whereas sulfapyridine (88%−ΔcGI at 4 µg/ml) is as effective as methotrexate, our positive control (88%−ΔcGI at 4 µg/ml).

**Conclusions:**

5-ASA inhibits MAP growth in culture. We posit that, unknowingly, the medical profession has been treating MAP infections since sulfasalazine's introduction in 1942. These observations may explain, in part, why MAP has not previously been identified as a human pathogen. We conclude that henceforth in clinical trials evaluating antiMAP agents in IBD, if considered ethical, the use of 5-ASA (as well as methotrexate and 6-MP) should be excluded from control groups.

## Introduction

In 1942 sulfasalazine (“Salazopyrin”) was introduced into clinical practice for ulcerative colitis. [1] Sulfasalazine has become, because of empirically observed clinical efficacy, “the most common medicine used to treat “Inflammatory” Bowel Disease (IBD) [2] with greatest efficacy in ulcerative colitis. [3]–[5]

Sulfasalazine is a conjugate of sulfapyridine and 5- aminosalicylic acid (5-ASA.) It is cleaved into its two component molecules following ingestion. [2] The sulfapyridine moiety [(2-(p aminobenzenesulphonamido) pyridine] is an acknowledged antibiotic. [6], [7] However, prevailing medical dogma concludes that “it is unlikely that (sulfasalazine's) antibacterial activity accounts for its clinical efficacy.” [2] In 1977, a two-week study on ulcerative proctitis, compared 5-ASA to sulfapyridine. Because of a decrease in inflammation in the 5-ASA group, the authors concluded, that in the therapy of ulcerative colitis the active moiety of sulfasalazine was 5-ASA. [8] As a consequence therapy with 5-ASA is called “anti-inflammatory” although “the mechanism of action of 5-ASA in IBD is uncertain.” [2]

Although controversial, there are increasingly compelling data that all [9], [10] or some of IBD may be caused by a single infectious agent *Mycobacterium avium* subspecies *paratuberculosis* (MAP.) [9]–[13] (& See [14] for review.) We have shown that two agents, methotrexate and 6-mercaptopurine (6-MP) [10], presumed to have “immunomodulatory” actions in IBD, [15], [16] are potent antiMAP antibiotics. We suggested that the decreases in pro-inflammatory cytokines in IBD “immunomodulatory” therapy might simply reflect a normal, secondary, physiological response, as the instigating MAP infection was effectively treated. We concluded that henceforth methotrexate and 6-MP should be excluded from the placebo group when evaluating antiMAP therapies in IBD. [10]

In this study we test the hypothesis that the “anti-inflammatory” action of 5-ASA and/or sulfapyridine could simply be due to one or both of sulfasalazine's components acting as an antiMAP antibiotic(s.) If correct the “anti-inflammatory” effect would simply represent a normal, secondary, physiological response as the causative MAP infection was controlled by antiMAP antibiotic action. Accordingly, in bacterial culture, we have evaluated the effect of sulfasalazine and individually and in combination its cleavage products 5-ASA and sulfapyridine, on *M. avium* including its subspecies MAP.

## Methods

This study was approved by the Research & Development Committee at the VAMC Bronx NY (0720-06-038) and was conducted under the Institutional Radioactive Materials Permit (#31-00636-07).

### Bacterial Culture:

In this study (as previously [10]) we use four well-characterized strains of mycobacteria. Two are MAP, a bovine isolate, ATCC 19698 (ATCC Rockville MD) and “Dominic” a human isolate from an individual with Crohn's disease (originally isolated by R. Chiodini [17].) The *M. avium* subspecies *avium* strains (hereinafter called *M. avium*) were ATCC 25291 (veterinary source) and *M. avium* 101. [18] Because it renders clinically resistant strains of MAP inappropriately susceptible to antimicrobials in cell culture, [19] we did not use the detergent Tween 80 (recommended to prevent mycobacterial clumping) in culture. Prior to inoculation cultures were processed as described. [10] [20]

Our negative control was intact sulfasalazine (a conjugate of sulfapyridine & 5-ASA) and the positive control was methotrexate. [10] Sulfapyridine and 5-ASA were evaluated individually and in combination (All from Sigma St Louis MO.) Aliquots of chemicals being evaluated were prediluted, stored at −80°C in 50 mM NaOH, thawed, used once and discarded. Volumes of NaOH were adjusted so that final concentration in each Bactec vial was always 3.2 mM NaOH. Agents were tested in serial dilutions from a minimum of 0.05 µg/ml to a maximum of 64 µg/ml (see individual Figures). When the individual molecules 5-ASA and sulfapyridine were studied in combination, the same weight for each was used as when tested individually (see appropriate Table.)

Data for the Bactec ® System (Becton-Dickinson Franklin Lakes NJ) are presented as cumulative growth index (cGI) units±SD (when necessary, see individual figures). cGI data for each experiment is presented until the day prior to any GI reading exceeding the assay limit of “999.” The effect (or lack thereof) of each agent is presented as the percent decrease in cGI units (%−ΔcGI), The calculation of %−ΔcGI is performed in two stages (using Excel) according to the formula:
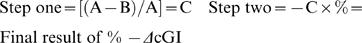
Where A = the cGI of the control inoculum for the given diluent (usually in these experiments NaOH); B = the cGI for the particular chemical at a particular dose being tested, incubated for the same number of days as A. C = the product of [(A−B)/A]. Raw data were archived onto Excel, collated and the cumulative results transferred to Prism (Graphpad, San Diego CA) for final graphing.

## Results

**Figure 1:**
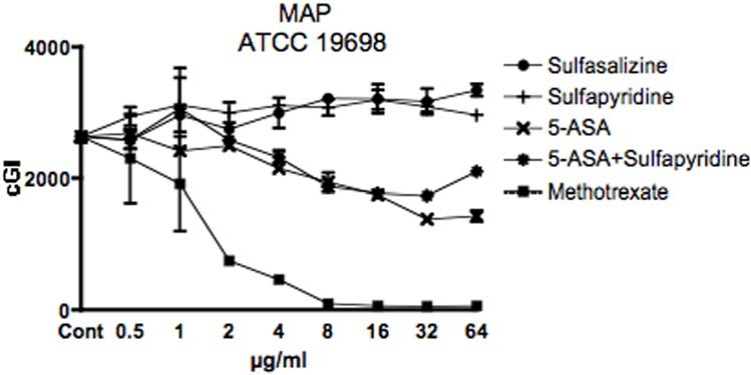
Cumulative GI data when 2.7×10^4^ CFU/vial of MAP ATCC 19698 was inoculated into each Bactec vial. Each drug dilution was studied in duplicate. Error bars are SD. The positive control is methotrexate, with maximal inhibition by 8 mg/ml. Neither the negative control sulfasalazine, nor sulfapyridine, have any inhibition. Both and 5-ASA alone and in combination with sulfapyridine have dose dependent inhibition.

**Figure 2:**
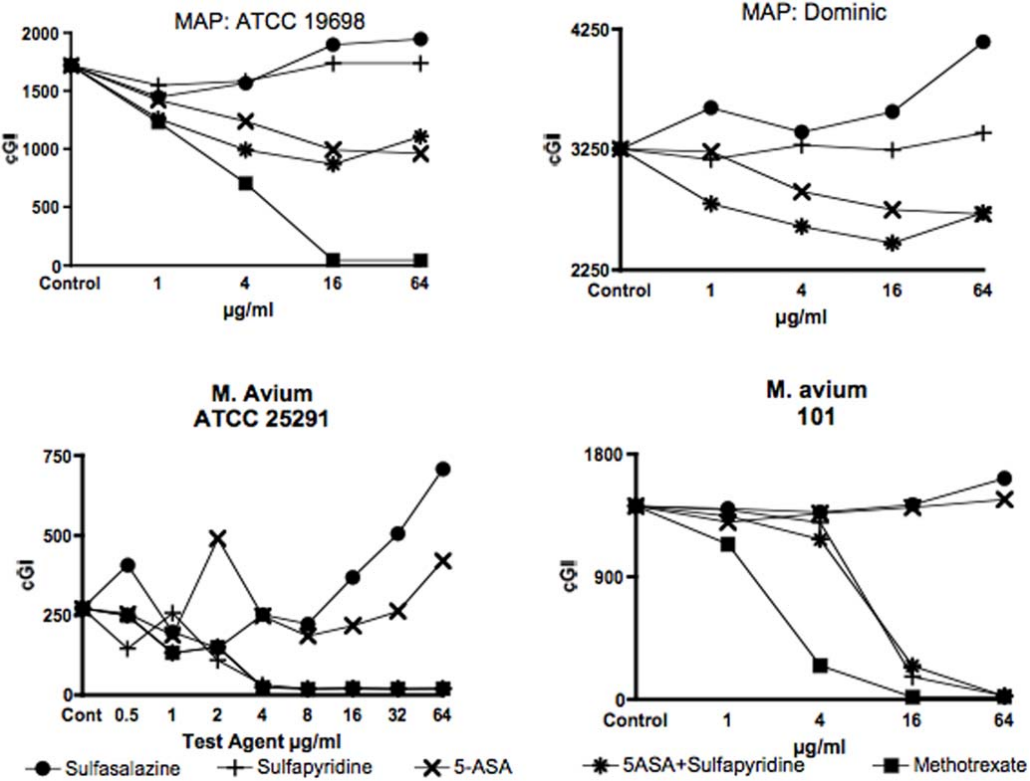
A composite graph of the two MAP and two M. avium bacterial strains studied. Each dose was studied in singlicate. For MAP ATCC 19698, 6×10^4^ CFU's were inoculated/vial. Sulfasalazine is the negative control for both MAP and *M. avium* strains. In all strains there is dose dependent increase in cGI. There is no inhibition by sulfapyridine alone with either MAP strain. 5-ASA has dose dependent inhibition on both MAP strains. Note the subtle synergy up to 16 µg/ml for both MAP strains for the 5-ASA+sulfapyridine group compared to 5-ASA alone. The positive control in the Dominic study was clarithromycin which, because it was diluted in methanol, is off scale. Accordingly, the clarithromycin data are not presented.

Data for the effect of test agents on bacterial growth are presented in two ways. Either as results for an individual mycobacterial strain (Figures 1 & 2) or as a comparison of the effect of each agent tested on all four mycobacterial strains in Tabular form, where inhibition is expressed as %−ΔcGI, and enhancement as % +ΔcGI (Tables 1–5.) Tables 1 & 2 are the experimental controls; Table 1 = the positive control methotrexate & Table 2 = the negative control sulfasalazine. Tables 3–5 have the agents tested, 5-ASA (Table 3), sulfapyridine (Table 4) and the combination of 5-ASA+sulfapyridine (Table 5.)

**Table 1:** Positive Control: Methotrexate

Methotrexate	MAP			M. avium	
	ATCC 19698		Dominic [10]	ATCC 25291	101
µg/ml	Fig. 1	Fig. 2	Fig. 2	Fig. 2	Fig 2
0.5	−13%			−44%	
1	−27%	−28%	−52%	52%	−20%
2	−72%			−72%	
4	−83%	−59%	−89%	−92%	−82%
8	−96%			−93%	
16	−98%	−97%	−93%	−92%	−99%
32	−98%			−94%	
64	−98%	−98%	−93%	−94%	−98%

%−ΔcGI

**Table 2 pone-0000516-t002:** Negative Control: Intact Sulfasalazine

Sulfasalazine	MAP			M. avium	
	ATCC 19698	Dominic		ATCC 25291	101
µg/ml	Fig. 1	Fig. 2	Fig. 2	Fig. 2	Fig 2
0.5	−2%			50%	
1	13%	−16%	10%	−27%	−1%
2	5%			−46%	
4	14%	−9%	4%	−7%	−3%
8	22%			−18%	
16	22%	10%	9%	36%	1%
32	20%			86%	
64	27%	13%	27%	160%	15%

%−ΔcGI

**Table 3 pone-0000516-t003:** Test Agent: 5-ASA

5-ASA	MAP			M. avium	
	ATCC 19698	Dominic		ATCC 25291	101
µg/ml	Fig. 1	Fig. 2	Fig. 2	Fig. 2	Fig. 2
0.5	2%			−6%	
1	−8%	−17%	−1%	−31%	−8%
2	−5%			80%	
4	−18%	−28%	−11%	−9%	−4%
8	−26%			−32%	
16	−33%	−42%	−16%	−20%	−1%
32	−47%			−3%	
64	−46%	−44%	−17%	55%	4%

%−ΔcGI

**Table 4 pone-0000516-t004:** Test Agent: Sulfapyridine

Sulfapyridine	MAP			M. avium	
	ATCC 19698	Dominic		ATCC 25291	101
µg/ml	Fig. 1	Fig. 2	Fig. 2	Fig. 2	Fig. 2
0.5	12%			−47%	
1	18%	−10%	−3%	−5%	−2%
2	14%			−60%	
4	18%	−8%	1%	−88%	−8%
8	17%			−93%	
16	21%	1%	0%	−93%	−88%
32	17%			−93%	
64	13%	1%	4%	−93%	−98%

%−ΔcGI

**Table 5:** Test Agents: 5-ASA+Sulfapyridine

5-ASA+Sulfapyridine	MAP			M. avium	
	ATCC 19698	Dominic		ATCC 25291	101
µg/ml	Fig. 1	Fig. 2	Fig. 2	Fig. 2	Fig. 2
0.5+0.5	−2%			−8%	
1+1	16%	−27%	−14%	−51%	−5%
2+2	−2%			−44%	
4+4	−12%	−42%	−20%	−91%	−17%
8+8	−29%			−93%	
16+16	−32%	−49%	−24%	−92%	−83%
32+32	−34%			−93%	
64+64	−20%	−35%	−16%	−92%	−98%

%−ΔcGI

The positive control is methotrexate, previously shown to be almost as effective as the acknowledged antiMAP antibiotic clarithromycin. [10] There is≥80%−ΔcGI at 4 µg/ml for all four species (Figures 1 & 2 and Table 1). In the MAP Dominic study (Figure 2), the positive control was clarithromycin, which because it was diluted in methanol was off scale, but showed maximal inhibition by 1 µg/ml (data not presented).

The negative control is the intact progenitor molecule sulfasalazine. [1] Sulfasalazine is manufactured by combining 5-ASA with sulfapyridine. Surprisingly, rather than having no effect or inhibiting *mycobacterial* growth we found that intact sulfasalazine actually enhances growth at high doses (Figures 1 and 2 & Table 2). By 64 µg/ml, sulfasalazine increases both MAP ATCC 19698 and Dominic by 27%+ΔcGI, (Figures 1 & 2 and Table 2) and of *M. avium* ATCC 25291 by 160%+ΔcGI (Figure 2 and Table 2.)

There are two different responses for MAP compared to *M. avium* from the two cleavage products of intact sulfasalazine, 5-ASA and sulfapyridine. Against MAP, 5-ASA has dose dependent inhibition. At 64 µg/ml of 5-ASA, inhibition against MAP ATCC 19608 is 46%-ΔcGI (Figures 1 & 2 and Table 3), and against Dominic inhibition is 17%-ΔcGI (Figure 2 and Table 3). In contrast, sulfapyridine alone has no inhibition against either MAP strain (Figures 1 and 2 and Table 4.)

In contrast to MAP, both *M. avium* strains are very susceptible to sulfapyridine. Inhibition is 88%-ΔcGI at 4 µg/ml for ATCC 25291 and 92%-ΔcGI at 4 µg/ml for M. avium 101 (Figure 2 and Table 4.) *M. avium* is not inhibited by 5-ASA, (Figure 2 and Table 4.)

Finally, the two components 5-ASA and sulfapyridine were studied in combination. For experimental comparability, equal doses of each agent were used (See Left Hand column in Table 5.) In two of the three MAP studies there is subtle evidence of synergy when the 5-ASA/sulfapyridine combinations are used (Figure 2 and Table 5.) This possible synergy is not seen in the MAP data presented in Figure 1. Different numbers of CFU's were inoculated in the two MAP ATCC 19698 studies. In the experiment from Figure 1 we inoculated 2.7×10^4^ CFU's and the experiment lasted 11 days and in Figure 2, 6×10^4^ CFU's were inoculated and the experiment lasted 10 days. There is no evidence of any 5-ASA/sulfapyridine synergy with either *M. avium* strain (Figure 2 and Tables 3–5.)

## Discussion

Although its precise mechanism of action has never been established, the utility of sulfasalazine (or 5-ASA alone) in the therapy of IBD, is uncontested since Svartz's seminal publication in 1942. [1] Sulfasalazine has been used because of empirical efficacy and prevailing medical dogma accepts that it acts in a non-specific “anti-inflammatory” manner. In the event that IBD is eventually accepted as being due to a MAP infection, our data are compatible with our hypothesis that the efficacy of 5-ASA is due to impairment of MAP growth.

Results with our positive control methotrexate, replicate our previous findings against all the *M. avium* strains studied. [10] In this study, our negative control is the intact progenitor molecule sulfasalazine that comprises sulfapyridine linked to 5-ASA. We show it has virtually no inhibitory action, and at high doses intact sulfasalazine actually enhances growth in all the strains studied. The mechanism(s) whereby mycobacteria are able to utilize uncleaved sulfasalazine remain to be elucidated.

We show completely different responses to 5-ASA and sulfapyridine in the two *M. avium* subspecies studied. Against MAP, 5-ASA is inhibitory in a dose dependent manner, whereas sulfapyridine alone has minimal effect. Our data therefore offer a rational explanation for the empirical observation, made thirty years ago [8], showing that 5-ASA is more active than sulfapyridine in the therapy of ulcerative proctitis. As a consequence, we suggest that the “anti-inflammatory” effect of 5-ASA may simply represent a normal, physiological, secondary response as an instigating MAP infection is treated.

In contrast, we observe that sulfapyridine is as effective as the positive control methotrexate against *M. avium* subspecies *avium*, whereas 5-ASA has no effect. We conclude that unlike its utility in putative MAP infections, 5-ASA has no role to play in *M. avium* subspecies *avium* infections.

As is conventional in studies such as these, we evaluated each agent on an equal weight basis. The data show that against MAP, 5-ASA is not nearly as effective as methotrexate. Our data are therefore consistent with many decades of empirical clinical observation with 5-ASA and methotrexate. Neither 5-ASA nor methotrexate has traditionally been administered as an “antibiotic.” Their dosage has been determined by titration to clinical efficacy and limited by side effects. For sulfasalazine, the recommended dosage is ≤4 gm/day, as tolerated. [3] In contrast, for methotrexate the usual “low dose” [10] that is used to treat “inflammatory” diseases such as IBD is 25 mg/week. [21] This >1000 fold difference (28,000 mg/wk sulfasalazine compared to 25 mg/wk methotrexate) is compatible with our data both in this manuscript and previously [10] showing that methotrexate is, on a weight/weight basis, far more effective than 5-ASA at inhibiting MAP growth.

In the clinical therapy of IBD, either sulfasalazine or one of its components, 5-ASA is used. Sulfapyridine is not used alone. Our culture inhibition data offer a rational explanation for these empirically derived medication use patterns. We show a possible subtle synergy when sulfapyridine is added to 5-ASA at higher inoculation counts. These observations need to be replicated in more MAP strains, different CFU inoculation counts and other inhibition methods. [22] Additionally, to prevent the emergence of resistant strains, single antibiotic therapy is not acceptable in the therapy of other mycobacterial diseases such as leprosy. [23] We conclude that, unless there is a sulfapyridine allergy, it may be preferable to treat with the combination medication sulfasalazine in preference to 5-ASA alone.

Previously we documented the antiMAP action of the “immune-modulators” methotrexate and 6-MP. We now show that the “anti-inflammatory” 5-ASA likewise has antiMAP action. These medications are bedrock “immuno-modulatory” and “anti-inflammatory” therapies in IBD and other “inflammatory” and “auto-immune” diseases. Our data raise the reasonable concern that an infectious cause of IBD has been overlooked, simply because the infectious agent has unknowingly been treated since 1942. It is therefore possible that the “I” in “I”BD could stand for “Infectious” and that the causative organism may be MAP.

We conclude that, since the placebo groups were receiving active antiMAP agents, prior studies that evaluated antiMAP agents need to be revaluated. Clinicians who continue to opine that MAP is not zoonotic will now need to substantiate their position. To do so they will need to conduct trials that exclude agents that we show have antiMAP activity from the placebo group. However, the ethical implications of excluding these agents [24] (see particularly Note of Clarification on Paragraph 29 added by the World Medical Association General Assembly, Washington 2002) will need to be fully addressed when they design their protocols.

### Conclusions and Recommendations

We show that the “anti-inflammatory” agent 5-ASA, interferes with the growth of MAP, an organism that may be the etiological factor for some, or all of IBD. Some of the implications of these observations are discussed.
